# Evolutionary history of *Wolbachia *infections in the fire ant *Solenopsis invicta*

**DOI:** 10.1186/1471-2148-5-35

**Published:** 2005-05-31

**Authors:** Michael E Ahrens, Dewayne Shoemaker

**Affiliations:** 1 Department of Entomology, 643 Russell Labs, 1630 Linden Drive, University of Wisconsin, Madison, WI 53706 USA

## Abstract

**Background:**

*Wolbachia *are endosymbiotic bacteria that commonly infect numerous arthropods. Despite their broad taxonomic distribution, the transmission patterns of these bacteria within and among host species are not well understood. We sequenced a portion of the *wsp *gene from the *Wolbachia *genome infecting 138 individuals from eleven geographically distributed native populations of the fire ant *Solenopsis invicta*. We then compared these *wsp *sequence data to patterns of mitochondrial DNA (mtDNA) variation of both infected and uninfected host individuals to infer the transmission patterns of *Wolbachia *in *S. invicta*.

**Results:**

Three different *Wolbachia *(*wsp*) variants occur within *S. invicta*, all of which are identical to previously described strains in fire ants. A comparison of the distribution of *Wolbachia *variants within *S. invicta *to a phylogeny of mtDNA haplotypes suggests *S. invicta *has acquired *Wolbachia *infections on at least three independent occasions. One common *Wolbachia *variant in *S. invicta *(wSinvictaB) is associated with two divergent mtDNA haplotype clades. Further, within each of these clades, *Wolbachia*-infected and uninfected individuals possess virtually identical subsets of mtDNA haplotypes, including both putative derived and ancestral mtDNA haplotypes. The same pattern also holds for wSinvictaA, where at least one and as many as three invasions into *S. invicta *have occurred. These data suggest that the initial invasions of *Wolbachia *into host ant populations may be relatively ancient and have been followed by multiple secondary losses of *Wolbachia *in different infected lineages over time. Finally, our data also provide additional insights into the factors responsible for previously reported variation in *Wolbachia *prevalence among *S. invicta *populations.

**Conclusion:**

The history of *Wolbachia *infections in *S. invicta *is rather complex and involves multiple invasions or horizontal transmission events of *Wolbachia *into this species. Although these *Wolbachia *infections apparently have been present for relatively long time periods, these data clearly indicate that *Wolbachia *infections frequently have been secondarily lost within different lineages. Importantly, the uncoupled transmission of the *Wolbachia *and mtDNA genomes suggests that the presumed effects of *Wolbachia *on mtDNA evolution within *S. invicta *are less severe than originally predicted. Thus, the common concern that use of mtDNA markers for studying the evolutionary history of insects is confounded by maternally inherited endosymbionts such as *Wolbachia *may be somewhat unwarranted in the case of *S. invicta*.

## Background

Innumerable insects and other terrestrial arthropods are infected with maternally transmitted endosymbionts. While many endosymbionts spread by increasing the fitness of their hosts, others spread by manipulating host reproduction in ways that specifically enhance transmission of infected cytoplasm, even if this results in reduced transmission of nuclear genes [1]. In these latter cases, such symbionts act as parasites. Parasitic endosymbionts are extremely prevalent in nature, and include many bacteria in the genus *Wolbachia *[2-4]. These endosymbiotic bacteria infect a wide variety of arthropods and filarial nematodes [2-4]. Although *Wolbachia *infecting filarial nematodes generally are considered mutualists, most *Wolbachia *strains infecting insects act as parasites. Recent surveys suggest that *Wolbachia *infect a substantial proportion of insect species, with estimates ranging from 17% [5-7] to 76% [8]. Extrapolation of these estimates suggests that millions of insect species are currently infected with *Wolbachia*, making these bacteria among the most widespread parasites on earth.

*Wolbachia *transmission within host species mainly occurs maternally through the egg cytoplasm, and as such, these microbes have evolved several mechanisms to enhance their own transmission that either increase their host's investment in daughters or decrease the reproductive success of uninfected females. These mechanisms include cytoplasmic incompatibility (CI), thelytokous parthenogenesis, feminization of genetic males, and male-killing [for recent reviews see [1,9,10]]. In addition to their vertical (maternal) transmission from mother to offspring, several independent lines of evidence clearly show *Wolbachia *are also horizontally transmitted both within and among different host species [3,11-18]. However, despite knowledge that *Wolbachia *can be transmitted horizontally, a general understanding of the frequency and mode of horizontal transmission within natural host populations is poorly documented.

One approach often employed to infer the transmission patterns and evolutionary history of *Wolbachia *infections within a given host species is to compare patterns of *Wolbachia *and host mtDNA genetic variation [19-40]. If the two genomes are strictly co-transmitted vertically from mother to offspring as predicted, then there should be strong linkage between a host's mtDNA genome and the associated *Wolbachia *genome. Depending on the age of infection, such linkage should be observable in patterns of molecular variation of the two genomes such that a given *Wolbachia *strain is associated with a particular mtDNA haplotype or clade of haplotypes [24,30,38,41-47]. On the other hand, this tight association is lost if horizontal transmission of *Wolbachia *occurs, in which case one would not necessarily expect concordant patterns of variation between the two genomes. As an example of using this approach, extensive studies of *Drosophila simulans *have revealed that this species is infected with at least four genetically distinct strains of *Wolbachia*, presumably representing four independent invasions across three distinct clades of mitochondrial haplotypes [26,30-37,44].

Several studies have been conducted examining the distribution and prevalence of *Wolbachia *infections among native South American populations of the fire ant *Solenopsis invicta*, as well as the effects of *Wolbachia *on mtDNA variation in this species. The general findings of these previous studies were: 1) the prevalence of *Wolbachia *infections varies significantly among different native geographic populations of *S. invicta*, 2) two divergent mtDNA haplotype lineages and two *Wolbachia *variants occur within *S. invicta*, and 3) a strong association between each *Wolbachia *variant and host mtDNA lineage exists, albeit these latter two conclusions were based on a relatively small number of samples from only two populations [38,46,48]. Interestingly, despite the apparent strong association between genomes, as well as evidence for a high fidelity of maternal transmission of *Wolbachia *within colonies of *S. invicta *in the field, Shoemaker et al. [46] found no consistent correlation between the presence of *Wolbachia *and either levels or patterns of mtDNA diversity. That is, levels of mtDNA variation in *Wolbachia*-infected and uninfected populations were similar and patterns of mtDNA variation within *Wolbachia*-infected populations did not differ consistently from neutral expectations, despite the prediction that strong positive selection acting on *Wolbachia *influences the evolutionary dynamics of other cytoplasmic genomes [46]. There are three potential non-mutually exclusive explanations for these puzzling results: 1) *Wolbachia *infections in *S. invicta *are sufficiently ancient so that levels of mtDNA variation have re-equilibrated to their levels prior to invasion of *Wolbachia*, 2) *Wolbachia *infections are horizontally transmitted within *S. invicta *such that the two genomes are not strictly co-transmitted as previously suggested, or 3) the evolutionary history of *Wolbachia *infections within *S. invicta *involves multiple independent invasions of one or more *Wolbachia *variants.

The major goal of the present study was to infer the transmission patterns and evolutionary history of *Wolbachia *infections within *S. invicta*. To accomplish our objective, we generated sequence data from two portions of the *Wolbachia *genome present in numerous infected individuals of *S. invicta *collected throughout the species' native range and subsequently compared these data to patterns of mtDNA variation to determine the extent of *Wolbachia *strain variation as well as the predominant mode of *Wolbachia *transmission in this species. In addition, we also use these data to address the issue of whether or not the significant variation in *Wolbachia *prevalence among fire ant populations is simply due to the presence of different *Wolbachia *variants in these populations. As we show below, our results based on these extensive sequence data lead to new insights regarding the history of *Wolbachia *infections in *S. invicta*, and in so doing, partly explain the paradoxical findings of previous studies on these ants.

## Results and discussion

### Diversity of *Wolbachia *strains in *S. invicta*

Our *Wolbachia *(*wsp*) sequence data, which includes partial *wsp *sequences from 138 *Wolbachia*-infected individuals, revealed only three unique variants within *S. invicta*. All three variants are identical to previously reported *Wolbachia *(*wsp*) variants from fire ants and fall into one of the two divergent major *Wolbachia *subgroups comprising *Wolbachia *strains specific to New World ants (*InvA *and *InvB*) [49,50]. Two of the variants were identical to *Wolbachia *variants previously reported to infect *S. invicta *(wSinvictaA and wSinvictaB; *InvA *and *InvB *subgroups, respectively), whereas the third "new" variant is identical to a variant previously reported to infect the closely related fire ant species *S. richteri *(wSrichteriA; *InvA *subgroup) [38].

Additionally, the *Wolbachia *16S sequence data from a subset of infected individuals did not reveal any new *Wolbachia *variants within *S. invicta*. The 16S sequences from all individuals infected with the variants wSinvictaA and wSrichteriA (based upon *wsp *sequences) were identical to each other as were the 16S sequences from individuals infected with wSinvictaB. However, the 16S sequences from individuals infected with the variants wSinvictaA and wSrichteriA differed by a single nucleotide substitution from those in individuals infected with wSinvictaB. All 16S sequences belong to the A group of *Wolbachia *(as opposed to the A and B groups for *wsp *sequences). This discrepancy between the two genes most likely results from an historical recombination event within the *Wolbachia *genome, which perhaps is not unexpected given previous studies showing recombination of *Wolbachia *genomes commonly occurs [51,52].

### Transmission patterns of *Wolbachia *in *S. invicta*

**Figure 1:**
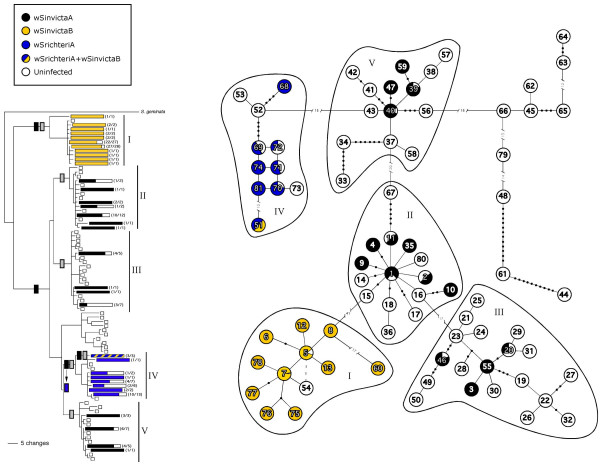
**Bayesian phylogenetic tree (A) and minimum spanning network (B) of mtDNA haplotypes from *S. invicta. ***Both the Bayesian phylogenetic tree and minimum spanning network of mtDNA haplotypes from *S. invicta *reprinted from Ahrens et al. [53]. Haplotypes associated with the three *Wolbachia *variants in *S. invicta *are indicated by coloured bars/circles. For each mtDNA haplotype, the coloured areas of bars/circles are proportional to the number of *Wolbachia*-infected individuals, also indicated by the values in parentheses. The five haplotype clades in the Bayesian tree harbouring *Wolbachia *infected individuals are linked to their corresponding haplotype clusters by Roman numerals I-V. Purported invasion/horizontal transmission events of *Wolbachia *into *S. invicta *under scenarios 1 and 2 are indicated by the grey and black coloured bars, respectively, on the Bayesian tree. Also indicated is the evolutionary transition of variant wSinvictaA to variant wSrichteriA (black box to blue box). See text for more details.

**Figure 2:**
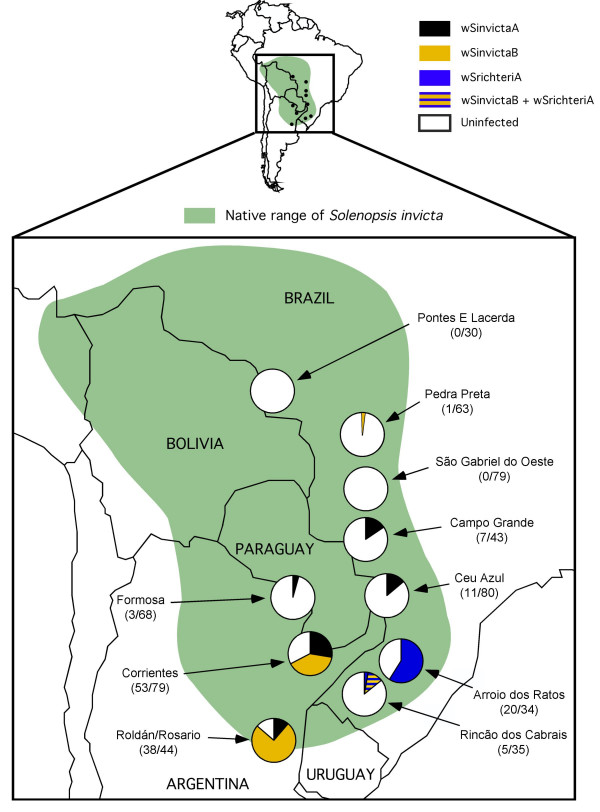
**Distribution and prevalence of *Wolbachia *variants in eleven sampled populations of *S. invicta. ***Each pie diagram shows the proportions of *Wolbachia*-infected (separately for each variant) and uninfected individuals in each geographic population (sample sizes in parentheses). The native range of *S. invicta *as currently understood is indicated by green shading and is based on Buren et al. [63], Trager [57], and Pitts [64].

Both *Wolbachia *(*wsp*) and mtDNA sequence data were available for 133 of 138 infected individuals (Table 1 and Figure 1): MtDNA sequence data were lacking for the remaining five *Wolbachia*-infected individuals, which are excluded from the comparative analyses below. The distribution of all mtDNA haplotypes within each of the eleven populations is shown in Table 2 and the particular haplotypes that are associated with *Wolbachia *infections is shown in Table 2 and Figure 1. A comparison of *wsp *and mtDNA sequence variation suggests a complex evolutionary history of *Wolbachia *infections in *S. invicta*, involving multiple independent invasions of *Wolbachia *into *S. invicta *followed by frequent secondary loss of infections in different maternal lineages. Indeed, these data suggest that at least six independent invasions involving three different *Wolbachia *variants have occurred into *S. invicta *(scenario 1 of Figure 1). The variant wSinvictaB apparently invaded *S. invicta *on two separate occasions. One of these invasions is most likely a rather recent event, as it is associated with only three individuals, all of which harbour an identical mtDNA haplotype (haplotype #51; incidentally, all three individuals also are infected with the wSrichteriA variant). The other invasion of wSinvictaB into *S. invicta *is presumably more ancient as evidenced by the strong association of this variant with a highly divergent mtDNA clade comprising closely related mtDNA haplotypes (i.e., clade I in Figure 1). There appears to have been a single invasion of the wSrichteriA variant into *S. invicta*, as its presence is limited to a single clade of mtDNA haplotypes (clade IV), all of which come from individuals collected from two populations in southern Brazil: Arroio dos Ratos and Rincão dos Cabrais (Figure 2; see Ahrens et al. [53]). Finally, the association of *Wolbachia *variant wSinvictaA with three highly divergent clades of mtDNA haplotypes (clades II, III, and V) is consistent with three separate, rather ancient invasions of this *Wolbachia *strain into *S. invicta*.

**Table 1:** Prevalence of *Wolbachia *variants in eleven sampled populations of *S. invicta*. N represents the number of individuals of *S. invicta *surveyed for *Wolbachia*. The total number of infected individuals is represented by n_inf _whereas n_*wsp *_and n_16S _represent the number of individuals for which the *wsp *and 16S genes, respectively, were sequenced. The data in column "*wsp *Strains" indicate the *Wolbachia *variants present in each population (based on *wsp *sequences) as well as the number of individuals infected with each variant (in parentheses). The number of individuals from each population where mtDNA sequence data were available is also indicated.

**City**	**Country**	**N**	**n_inf_**	**n_*wsp*_**	***wsp *Strains**	**n_16S_**	**mtDNA**
Corrientes	Argentina	79	53	53	wSinvictaA (22), wSinvictaB (31)	6	54
Formosa	Argentina	68	3	3	wSinvictaA (2)	1	38
Roldán	Argentina	14	13	13	wSinvictaA (1), wSinvictaB (12)	5	14
Rosario	Argentina	30	25	25	wSinvictaA(4), wSinvictaB (21)	2	29
Arroio dos Ratos	Brazil	34	20	20	wSrichteriA (20)	6	33
Rincão dos Cabrais	Brazil	35	5	5	wSrichteriA (1), wSrichteriA+wSinvictaB (4)	1	10
Campo Grande	Brazil	43	7	7	wSinvictaA (7)	2	29
Ceu Azul	Brazil	80	11	11	wSinvictaA (11)	3	66
Pontes E Lacerda	Brazil	30	0	0	-	0	28
Pedra Preta	Brazil	63	1	1	wSinvictaB (1)	1	48
São Gabriel do Oeste	Brazil	79	0	0	-	0	51
	**Totals:**	**555**	**138**	**138**		**27**	**400**

**Table 2:** Distribution of different mtDNA haplotypes within the eleven sampled populations of *S. invicta*. *h *represents the number of different mtDNA haplotypes occurring in each population (see Table 1 for total number of mtDNA sequences generated from individuals of each population). Haplotypes occurring in more than one population are underlined, and haplotypes found in *Wolbachia*-infected individuals are in bold italics.

**City**	**Country**	***h***	**Haplotypes Occurring in Populations**
Corrientes	Argentina	20	***1***, ***2***, ***3***, ***4***, ***5***, ***6***, ***7***, ***8***, ***9***, ***10***, ***11***, ***12***, ***13***, 14, 15, 16, 17, ***35***, 54, 55
Formosa	Argentina	18	18, 19, ***20***, 21, 22, 23, 24, 25, 26, 27, 28, 29, 30, 31, 32, 33, 34, 36
Roldán	Argentina	5	***1***, ***7***, ***75***, ***76***, ***77***
Rosario	Argentina	6	***1***, ***5***, ***7***, ***78***, 79, 80
Arroio dos Ratos	Brazil	7	***69***, ***70***, ***71***, ***72***, 73, ***74***, ***81***
Rincão dos Cabrais	Brazil	5	***51***, 52, 53, 67, ***68***
Campo Grande	Brazil	5	***46***, ***47***, 48, 49, 50
Ceu Azul	Brazil	11	37, 38, ***39***, ***40***, 41, 42, 43, 56, 57, 58, ***59***
Pontes E Lacerda	Brazil	6	45, 62, 63, 64, 65, 66
Pedra Preta	Brazil	3	44, ***60***, 61
São Gabriel do Oeste	Brazil	2	48, 49

An alternative scenario, however, is that there have been only three independent invasions of *Wolbachia *into *S. invicta*: Two of these invasions involve wSinvictaB (as described above) whereas the third invasion involves *Wolbachia *variant wSinvictaA (scenario 2 of Figure 1). Under this scenario such a single invasion of wSinvictaA into *S. invicta *presumably would have to be quite ancient, since it requires that the infection would have had to be present in the common ancestor of clades II-V (see Figure 1). Assuming a divergence rate of 2% per million years [54], our estimate of the net average nucleotide divergence among all mtDNA haplotypes comprising clades II-V (2.4%; Ahrens et al. [53]) would suggest this invasion of wSinvictaA (or most recent *Wolbachia *sweep) occurred roughly 1.2 mya. An additional caveat of this scenario is that wSrichteriA is not a novel, independently acquired *Wolbachia *infection but instead represents a derived variant of wSinvictaA (see Figure 1).

Although these rather restrictive conditions might lead one to conclude that a single invasion of wSinvictaA into *S. invicta *seems unlikely, this is not necessarily the case. First, whilst it is tempting to interpret the high levels of divergence among mtDNA haplotypes as indicating an ancient invasion of *Wolbachia*, one must be cautious when using estimates of mtDNA sequence divergence for inferring evolutionary rates simply because such high divergence may be the result a *Wolbachia*-driven increase in mtDNA substitution rates [39]. If true, then we may have substantially overestimated the time of invasion of wSinvictaA into *S. invicta*. A necessary requirement, however, is that individuals comprising the separate clades correspond to different lineages or populations that themselves are connected by little or no migration, since significant gene exchange would erase the signature of high divergence among clades. Otherwise, the most plausible explanation for the association of wSinvictaA with these divergent clades is that multiple independent invasions have occurred into *S. invicta *(i.e., scenario 1 above). For most populations currently infected with wSinvictaA, the condition of substantially reduced gene flow among populations holds: Ahrens et al. [53] found that genetic divergence among populations is very high and that mtDNA genetic variation is correlated with geography such that 76 out of 81 mtDNA haplotypes identified in *S. invicta *were exclusive to single populations. Thus, the likelihood of a single invasion seems much more reasonable when we consider not only the possible effects of *Wolbachia *within populations but also how *Wolbachia *infections can accelerate divergence among populations (divergence among mtDNA lineages), especially those connected by very limited gene flow. Finally, we should also point out that the additional above requirement that wSrichteriA is a derived variant of wSinvictaA also is quite reasonable given that these two strains differ by only a single nucleotide substitution at the highly evolving *wsp *gene.

Regardless of the presumed number of invasions of *Wolbachia *into *S. invicta *(three, six, or perhaps more), it is clear that the secondary loss of *Wolbachia *infections from host lineages following invasion is very common. Such frequent loss of infections is most obvious when one considers the fact that uninfected individuals harbour both derived mtDNA haplotypes and ancestral haplotypes inferred to be associated with the original infection (Figure 1). Previously, Shoemaker et al. [48] estimated that the fidelity of maternal transmission of *Wolbachia *in *S. invicta *in nature generally is very high (>99%), but nonetheless is not perfect, ranging from 90–100% within different matrilines.

Thus, our data partly resolve the paradox of a lack of a consistent correlation between the presence of *Wolbachia *and either levels or patterns of mtDNA diversity in *S. invicta*. Clearly, the previous assertion of strictly vertical transmission of *Wolbachia *in fire ants breaks down upon finer-scale analysis. Multiple independent invasions of *Wolbachia *into *S. invicta *have occurred, and in every case these have been followed by frequent secondary loss of infections. Thus, although we predicted a strong association between the mtDNA and *Wolbachia *genomes since both are co-transmitted from mother to offspring, the strong association of the two genomes in fire ants clearly has broken down over time due to frequent horizontal transmission and secondary loss of *Wolbachia *strains [26,36,44-46,55].

### *Wolbachia *distribution and prevalence in *S. invicta*

Variation in the distribution and prevalence of *Wolbachia *in natural populations of *S. invicta *may be due to: 1) presence of different *Wolbachia *variants within and among populations 2) genetic differences among host individuals from different populations or 3) genetic drift [48]. To attempt to address this issue, we examined the *Wolbachia *strain identities and their corresponding frequencies within each of the eleven sampled populations of *S. invicta*. If variation in *Wolbachia *prevalence is due simply to differences in the particular *Wolbachia *variants or combinations of variants within and among these populations, then we might expect that despite differences in overall *Wolbachia *prevalence among populations the prevalence of any particular *Wolbachia *variant is similar in each of the host populations where it occurs. Thus, a simple explanation for the observed variation in prevalence may be that the array of *Wolbachia *variants differs among host populations. On the other hand, if this variation results from genetic differences among host individuals from different populations, then one might expect that the prevalence of specific *Wolbachia *variants varies among different host populations, and possibly that the variants are associated with quite different mtDNA haplotypes in each population. Although the effects of *Wolbachia *on *S. invicta *are currently unknown, we would expect infection prevalence to vary stochastically if *Wolbachia *do not have any measurable fitness or sex ratio effects on their fire ant hosts.

The distribution and prevalence of the three *Wolbachia *variants within the eleven sampled populations of *S. invicta *is shown in Figure 2. The wSinvictaA variant occurs at similar prevalence (11.4–27.8% of individuals) in four of the five populations where it is found, possibly indicating this low prevalence represents the stable equilibrium frequency of this variant. The similar prevalence of wSinvictaA in different populations that are both genetically differentiated and separated by great geographical and ecological differences [53] suggests that the dynamics and prevalence of this variant are most likely not strongly affected by its host or environment. The wSinvictaB variant is largely confined to individuals collected from the southwestern populations of Corrientes and Roldán/Rosario. This variant occurs at relatively high prevalence in these populations (39.2–75.0%). Finally, the wSrichteriA variant has a very restricted distribution and is found only in individuals from the Arroio dos Ratos and Rincão dos Cabrais populations in the southernmost portion of Brazil. Our survey data revealed that 36% of all colonies surveyed from these populations harbour this *Wolbachia *variant.

Together, these data indicate that the variation in *Wolbachia *prevalence among populations can be explained largely by differences in the array of *Wolbachia *variants within host populations. Even so, we cannot discount completely a role for host effects in determining *Wolbachia *prevalence given the very high levels of genetic differentiation among populations [46,53,56]. Additionally, while our data do not imply an obvious role for environmental conditions affecting *Wolbachia *dynamics, it is interesting to note the apparent positive correlation between *Wolbachia *prevalence and latitude. An analogous pattern previously has been reported for a *Wolbachia *variant infecting the beetle *Chelymorpha alternans*. In this host species, *Wolbachia *prevalence apparently is lower in areas experiencing longer dry seasons and higher average daily temperatures [24]. Thus, although unlikely, it remains possible that the overall *Wolbachia *infection dynamics in *S. invicta *are influenced by differences in environmental conditions as well, with higher *Wolbachia *prevalence occurring in the more southerly temperate populations.

Finally, our results combined with mtDNA data from earlier studies argue against the previous hypothesis that variation in *Wolbachia *prevalence is simply due to the recent invasion and ongoing spread of *Wolbachia *in *S. invicta*. First, a substantial number of polymorphic sites were found in the mtDNA sequences comprising each of five clades (I-V), indicating the *Wolbachia *infections are sufficiently ancient enough that numerous mtDNA mutations have accumulated since the most recent invasion(s) of *Wolbachia*. Assuming a divergence rate of 2% per million years [54], estimates of the average sequence divergence among mtDNA haplotypes within clades I-V (0.1–1.2%; Ahrens et al. [53]) would suggest the most recent invasion of *Wolbachia *(or most recent *Wolbachia *sweep) within any of these clades roughly occurred at least 50,000 years BP (Although *Wolbachia *endosymbionts may accelerate divergence between lineages or populations, recurrent *Wolbachia *sweeps have the opposite effect on differentiation within populations and result in substantially reduced mtDNA variation within populations [30,39]). The finding that the composition and diversity of mtDNA haplotypes found in infected and uninfected individuals within populations are virtually identical clearly suggests that uninfected individuals are derived from infected lineages via incomplete maternal transmission of *Wolbachia *and lends further support to the hypothesis that *Wolbachia *infections in *S. invicta *are evolutionarily old. An alternative possibility, which we consider less likely, is that there has been rampant horizontal transmission of the same *Wolbachia *variants within and among *S. invicta *populations.

## Conclusion

The evolutionary history of *Wolbachia *in *S. invicta *is far more complex than previously recognized: at least three and possibly as many as six horizontal transmission events involving three different variants have occurred into *S. invicta*. Further, in every case these independent acquisitions of *Wolbachia *have been followed by multiple independent losses of *Wolbachia *infections over time. Indeed, we should note that if loss of *Wolbachia *infection occurs as commonly as our data suggest, then we likely have underestimated the number of invasions or horizontal transmissions of *Wolbachia*. These extensive sequence data also suggest that the significant variation in *Wolbachia *prevalence among fire ant populations most likely is due simply to the presence of different variants limited to specific regions of *S. invicta's *range, but roles for both host effects and the environment in accounting for the observed patterns cannot be excluded. Our results also partly explain the previous puzzling findings of no clear effects of *Wolbachia *infection on patterns of mtDNA variation and substitution in fire ants [46]. *Wolbachia *transmission over evolutionary time appears to be uncoupled from that of the mtDNA genome such that the predicted effect of *Wolbachia *in reducing host mtDNA variation is not clearly evident as originally predicted. Thus, our previous concern that recurrent *Wolbachia *sweeps within fire ant populations may confound the use of mtDNA markers for studying the evolutionary history of fire ants (i.e. phylogeographic studies, identification of source populations), as the invasion of new strains would erase all pre-existing variation, seems somewhat unwarranted.

On the other hand, the high levels of divergence among mtDNA haplotype clades (~3.2% [53]) are analogous to patterns reported for the two *Wolbachia*-infected insect species, *Drosophila recens *and *D. simulans*, and may be the footprint of another predicted effect of *Wolbachia *infections, namely, an accelerated mtDNA substitution rate as a result of recurrent *Wolbachia *sweeps (see Shoemaker et al. [39] for full discussion). For example, Shoemaker et al. [39] observed an mtDNA-specific accelerated rate of evolution in *D. recens*, a species in which virtually all individuals are infected by a single *Wolbachia *strain, relative to the closely related uninfected species *D. subquinaria*. In *D. simulans*, previous studies have revealed that despite very little sequence variation within each of the three defined mtDNA haplotype clades, substantial differentiation exists among these clades [26,30-37,44]. Although no formal comparative analyses have been conducted in either *S. invicta *or *D. simulans *to test the above hypothesis, one possible explanation for the high level of divergence among these well-defined mtDNA haplotype clades in both species is that it results from *Wolbachia*-driven acceleration in the mtDNA substitution rate [39]. Together, these three studies lend support to the hypothesis that maternally-inherited endosymbiont infections may increase the rate of substitution in mtDNA [39]. Clearly, additional comparative studies in other insects are needed to test the generality of this hypothesis, especially since such effects have important consequences for the assumptions of neutrality and use of mtDNA as a molecular clock in insects.

## Methods

### Collection and identification of ants

Individuals of *S. invicta *were collected from native populations in Argentina and Brazil in 1992 and 1998 (Table 1). Multiple workers and winged virgin queens were collected from each of 555 colonies representing eleven geographic populations distributed over much of the known native range of *S. invicta *(see Figure 1 of Ahrens et al. [53] for locations). All collected individuals were identified as *S. invicta *by J. P. Pitts using species-informative morphological characters [57,58].

### Sequencing of *Wolbachia *strains

DNA was extracted from a single individual from each of the 555 colonies using the Puregene DNA isolation kit (Gentra Systems) [38,59]. We previously screened all 555 DNA extracts for the presence of *Wolbachia *by means of PCR using the primers *wsp*81F and *wsp*691R [48,59,60]. These *wsp *primers amplify a portion of a highly-variable gene encoding the *Wolbachia *outer surface protein [59,60]. Our previous survey of *S. invicta *revealed that 138 of the 555 individuals (colonies) were *Wolbachia*-infected (see Table 1). For the present study, we sequenced a portion of the *wsp *gene from all 138 infected individuals using the above primers. *Wolbachia *DNA was PCR-amplified in 30-μL volumes, with the PCR reaction components and thermal cycling conditions identical to those described in Shoemaker et al. [38]. *Wsp *PCR amplicons were purified for sequencing using Ampure magnetic beads (Agencourt Bioscience Corp.) and subsequently used directly in standard fluorescent cycle-sequencing PCR reactions (ABI Prism Big Dye terminator chemistry, Applied Biosystems). Sequencing reactions were purified using CleanSEQ magnetic beads (Agencourt Bioscience Corp.) and run on an ABI 3700 sequencer at the UW Biotechnology Center DNA Sequencing Laboratory.

Initial sequencing results of the *wsp *gene revealed the presence of more than one *Wolbachia *strain in three individuals of *S. invicta *(i.e., multiple peaks or frameshifts in electropherogram profiles were observed). For these three individuals, *Wolbachia *DNA was PCR-amplified as described above, except the final extension at 72°C was increased to 30 minutes. PCR amplicons were cloned directly into a vector following manufacturer's suggestions (Topo TA cloning kit, Invitrogen corp.) and resulting colonies screened for the presence of the desired *wsp *PCR insert using the *wsp *primers. For each individual, PCR-amplified products from ten colonies (which presumably had the *wsp *insert) were purified and sequenced as described above.

We also PCR-amplified and sequenced a 945 base portion of the *Wolbachia *16S gene using primers specific to this region [3] from a subset of the infected individuals within each population in an attempt to further characterize and identify unique *Wolbachia *strains (27 sequences total). PCR reaction components and thermal cycling conditions were identical to those described in O'Neill et al. [3]. Purification and sequencing of 16S amplicons, as well as cloning and sequencing of individuals possessing more than one *Wolbachia *strain, were carried out as described for the *wsp *gene above.

### Comparing *Wolbachia *(wsp) and mtDNA variation

Both the phylogeny and minimum spanning network of 81 unique mtDNA haplotypes representing 400 individuals (colonies) from the eleven populations used in the present study were generated previously by Ahrens et al. [53] using MrBayes 3.0 [61] and ARLEQUIN ver. 2.000 [62], respectively. Both methods of analysis identified six well-supported clades (clusters) of closely related mtDNA haplotypes, with each clade separated from the others by at least 18 mutational steps. With few exceptions, each clade is comprised of mtDNA haplotypes present in individuals from only one or two geographically proximal populations of *S. invicta *[for a more detailed description, see [53]]. For the present study, we used our *wsp *gene sequence data to determine the infection status, infection frequency, and strain identity for individuals of each mtDNA haplotype within the pre-existing networks.

## Authors' contributions

MEA carried out the majority of the molecular work and performed phylogenetic data analyses. DDS designed and coordinated the study, collected all of the ants used for the study, carried out a portion of the molecular work, and performed most of the data analyses. Both authors contributed to writing the manuscript and approved the final manuscript.
